# Supplementary material for: Determinants of the Pace of Global Innovation in Energy Technologies
Source: PLoS One. 2013 Oct 14;8(10):e67864. doi: 10.1371/journal.pone.0067864 (PMC3796528; doi:10.1371/journal.pone.0067864)
Supplement: File S1 — Supporting Information. (PDF) [file pone.0067864.s001.pdf]

Determinants of the pace of global innovation in energy technologies

Luís M. A. Bettencourt[*], Jessika E. Trancik[*+], Jasleen Kaur
[*]Contributed equally
[+]Correspondence to: trancik@mit.edu

Supporting Information

1. Materials and Methods

Patents dataset

We created a database of published energy technology patents and patent applications worldwide from keyword searches of Delphion (www.delphion.com). This system contains patent documents from sources worldwide since the early 1970s or the inception of the patent office. The main contributions in numbers of patents in energy technologies are the Patent Abstracts of Japan (patent application information available since 1976) from the Japan Patent Information Organization (Tokyo, Japan); the United States Patent and Trademark Office (Washington, DC, USA, complete information about granted patents since 1974, and published patent applications since 2001); the European Patent Office (Vienna, Austria, granted patent information since 1980, applications since 1979); INPADOC, which contains "71 world patent signatories and legal status information from 42 patent offices" since 1968; the German Patent and Trademark Office (Munich, Germany, granted patents since 1968, applications since 1968); and the World Intellectual Property Organization (Geneva, Switzerland, information covering 175 countries since 1978).

Keywords searches were performed based on approaches outlined in earlier references (1, 2), with slight modifications that we found improved coverage and reduced the incidence of false positives. (There are bibliometric advantages of using keyword searches rather than patent codes or classes, including completeness, accuracy, and consistency across patent offices.) We specified terms for individual technologies: petroleum, natural gas, and coal, which make up fossil fuels; photovoltaics (referred to as 'solar' in the paper), hydroelectric, geothermal, wind, biofuels, which make up renewable technologies; and nuclear fission and fusion, which together make up nuclear technologies.

Patents for each technology were retrieved via the keyword searches (case insensitive):

Petroleum: (oil or gasoline or petroleum) and (electric* or energy or power or turbine) and (generat*)

Natural gas: ("natural gas") and (electric* or energy or power or generat* or turbine or vehicle)

Coal: "coal" and (electric* or energy or power or generat* or turbine or vehicle)

Solar: (photovoltaic or "photo voltaic" or (solar and cell)) and (electric* or energy or power or generat* or turbine or vehicle)

Hydroelectric: ("hydro electric" or "hydro power" or hydroelectric or hydropower) and (electric* or energy or power or generat* or turbine or vehicle)

Geothermal: (geothermal or "geo thermal") and (electric* or energy or power or generat* or turbine or vehicle)

Wind: "Wind" and (electric* or energy or power or turbine) and (generat*)

Biofuels: ("bio fuel" or "bio diesel" or biofuel or biodiesel) and (electric* or energy or power or generat* or turbine or vehicle)

Nuclear fission: (nuclear fission) and (electric* or energy or power or generat* or turbine or vehicle)

Nuclear fusion: (nuclear fusion) and (electric* or energy or power or generat* or turbine or vehicle)

Individual technologies were then aggregated to form the classes of fossil fuel, renewable and nuclear technologies, and ultimately the set of patents in energy technology.

The breakdown of numbers of patents published by technology are given in Table S1 and their shares by nation are illustrated in Fig. 2C. Figures in the paper refer to patents published unless otherwise noted (e.g. in Figure 4, which is based on patent applications).

Coal	14894	Fossil Fuels	30771
Petroleum	11601		
Natural Gas	5159		
Solar	21933	Renewables	41470
Wind	15279		
Hydroelectric	2462		
Geothermal	1928		
Biofuels	583		
Nuclear Fusion	904	Nuclear	1077
Nuclear Fission	179		

Table S1: Number of total patents per technology sector, up to the end of 2009. Note that a small number of patents may appear in more than one technological sector.

R&D Funding data sources

International Energy Agency (IEA) data on Public Funding

Data for public research and development (R&D) funding of selected energy technologies was obtained from the International Energy Agency (IEA). These estimates include demonstration projects. Figures are given in 2009$ (prices and exchange rates). Data are

available at http://wds.iea.org/WDS/ under the category "R&D budgets" (3). Due to lack of data, R&D funding from China was not included in the database and analysis.

Data on Private Funding
US private R&D funding for energy was obtained from the National Science Foundation's Division of Science Resources Statistics, Industrial Research and Development Information System (http://www.nsf.gov/statistics/iris/search_hist.cfm?indx=21), and from reference (4).

Data on several years (2006 and 2007) of private funding for global private R&D in clean energy was obtained from New Energy Finance http://www.newenergymatters.com. (Global private R&D funding for clean energy was approximately equal to that of global public R&D funding for 2006 and 2007.)

Data on Cumulative Production
Global production data for solar (photovoltaics) and wind was obtained from references (5-7). US coal electricity production data was obtained from reference (8). See Figures S8 and S10. These are the data used to populate the time series for *C*, as discussed below.

2. Derivation of production function for patents

We draw on the basic conceptual model of knowledge creation by Griliches (9), which relates patenting, *P*, to public R&D, *R*, and market size, *C*, via knowledge *K* (see Figure 3, main manuscript). We modify the conceptual model to capture the relationship between the proportional changes in variables, and express it as follows:

$$\frac{1}{P}\frac{dP}{dt} = \alpha\frac{1}{R}\frac{dR}{dt} + \beta\frac{1}{C}\frac{dC}{dt} + \delta\frac{1}{\Delta}\frac{d\Delta}{dt}, \qquad \text{(S1)}$$

which is eq. (2) in the main text, though there we do not show the component relating to Δ, as discussed below. We assume (and test against the data) that the coefficients α, β, δ are constants in time. Then we can integrate (S3) to obtain

$$P(t) = P_0 R^{\alpha}(t) C^{\beta}(t) \Delta^{\gamma}(t). \qquad \text{(S2)}$$

This is the form used below and in the main text in eq. (1) to estimate parameter values (where we absorb the Δ dependence in the other terms, see comments below) and verify that the ratios α, β are consistent with the hypothesis of their constancy. We also directly fit eq. (S1 and 2 in the main text) to the data.

A few comments on this derivation and its consequences are in order:

1) We have used cumulative quantities over time because the effects of knowledge production are nonlocal in time and accumulating. We find empirically that this is necessary to account for trends in the data. The treatment of these variables as cumulative quantities (stocks) is different from several commonly observed functions in the literature,

such as the Cobb-Douglas production function, in that even if an input ceases to grow the cumulative base in that input will continue to enhance the value of new investment in the others. In our model the effects of market driven investment in knowledge creation today multiply the cumulative past R&D investment and vice-versa. This is because of the character of knowledge, which differs from other produced goods and services to which a Cobb-Douglas production function (with non-cumulative inputs) typically applies. In practice, past knowledge can become obsolete but the rate at which this happens is difficult to quantify.

2) We assume that the quantities *R* and *C* can be expressed at a single time lag relative to *P*, which is a particular (simplifying) case in the integration from Eq. (S1) to (S2). It is only in the case of wind technologies that we observe a tangible lag, where *C* lags patents by 3-4 years. This may reflect investments that are made ahead of large, planned wind installations. These assumptions can be relaxed but only at the cost of introducing contributions at more time points, thus more functional freedom in the fit, and potentially over-fitting of the data.

3) The quantity Δ is not observed but it could account, for example, for venture capital. However, we find that any systematic *independent* temporal trend that it may introduce in patterns of invention is at most very small. An independent factor driving patents would be expected to lead to a temporal variation in P_0, which is not observed. This implies that its variation is either negligible or that it is strongly correlated, and can be expressed in terms of, temporal trends in *C,* and *R*, i.e.

$$\delta \frac{1}{\Delta}\frac{d\Delta}{dt} \propto a\delta \frac{1}{R}\frac{dR}{dt} + b\delta \frac{1}{C}\frac{dC}{dt} \qquad \text{(S3)}$$

In this case the *measured* exponents include an implicit contribution from variations in Δ as in $\alpha \to \alpha + a\delta, \quad \beta \to \beta + b\delta$. However, because we do not know *a*, *b* we can only assert that they must be approximately constant in time. Note, that in principle they can be negative, if the relative variation of Δ and the other variables were anti-correlated.

4) In the observed exponents α, β (absorbing the contributions discussed above) we see that $\alpha+\beta <1$, which holds empirically in all cases estimated below.

5) The multiplicative effects of knowledge creation on technology improvement, market expansion and presumably increased profits, implies that (when examined in the opposite direction from the one written above) $C \sim P^{1/\beta}$, where $1/\beta$ is larger than 1. This relationship shows increasing returns to scale in economic performance to knowledge creation (proxied by patents), as expected from general theoretical considerations. Once created, this virtuous cycle may lead to the self-sustaining improvement of a technology in tandem with its market expansion, as suggested in Figure 3.

3. Model regression and best-fit parameter estimates

In this section we fit equation 1 in the main text to the globally aggregated time series data for cumulative public research and development funding (R&D), production, and patents for solar, wind and coal. The results are shown in Figure 4 where we plot the natural logarithm of cumulative patent counts over time. To further verify these results with stationary time series, we also fit equation 2 in the main text to the data.

Solar	N	$logP_0$	std error	α	std error	β	std error	adj-R^2
**t_R=0, t_C=0**	**34**	**3.97**	**0.16**	**0.22**	**0.02**	**0.41**	**0.01**	**0.997**
t_R= 0, t_C=+1	33	3.46	0.17	0.27	0.02	0.41	0.01	0.996
t_R=-1, t_C=0	33	4.34	0.15	0.18	0.02	0.41	0.01	0.996
t_R=-1 t_C=-1	33	4.44	0.22	0.18	0.03	0.41	0.01	0.997

Table S2A: Results of applying Eq. (1) in the main text to cumulative patents filed globally in solar technologies, cumulative global R&D funding and cumulative global production (in terms of module capacity). Patents are aggregated by date of filing. The row highlighted indicates the best-fit parameters used in Figure 4 of the main text. Other rows show the sensitivity of the parameter estimates fits to different time lags, t_R for R&D and t_C for production, measured in years. ('+1' means that patents precede *C* or *R* by one year.) The parameter estimates are significant in all cases, especially for the best fit, which has p values of 2e-16, 1.65e-09 and 8.48e-10 for $log\ P_0$, α and β respectively.

Solar	N	α	std error	β	std error	adj-R^2
**t_R=0, t_C=0**	**34**	**0.22**	**0.03**	**0.35**	**0.02**	**0.850**

Table S2B: Results of applying Eq. (2) in the main text to the first difference of the logs of cumulative solar patents, R&D funding and production (in terms of module capacity). These time series pass the Dickey-Fuller test of stationarity. The row shown is the best-fit time lag (which is consistent with that in Table S2A). The parameter estimates are significant, with p values of 2.45e-8, and 1.27e-17 for α and β, respectively.

Wind	N	$logP_0$	std error	α	std error	β	std error	adj-R^2
t_R=-1, t_C=+3	22	2.36	0.62	0.28	0.11	0.37	0.03	0.981
t_R= 0, t_C=+3	23	2.67	0.79	0.23	0.14	0.38	0.04	0.981
t_R=-1, t_C=+4	21	1.71	0.57	0.40	0.10	0.32	0.03	0.980
**t_R= 0, t_C=+4**	**22**	**0.99**	**0.59**	**0.51**	**0.11**	**0.30**	**0.03**	**0.983**

Table S3A: Results of applying Eq. (1) in the main text to cumulative patents filed globally in wind technologies, cumulative global R&D funding and cumulative global production (in terms of turbine capacity). The highlighted row indicates the best-fit parameters used in Figure 4 of the main text. p-values for best fit parameters (highlighted) are 0.105, 7.84 e-5, 1.35 e-9, for $log\ P_0$, α and β respectively.

Wind	N	α	std error	β	std error	adj-R^2
**t_R=0, t_C=+4**	**22**	**0.48**	**0.13**	**0.29**	**0.06**	**0.10**

Table S3B: Results of applying Eq. (2) in the main text to the first difference of the logs of cumulative wind patents, R&D funding and production (in terms of turbine capacity). These time series pass the Dickey-Fuller test of stationarity. The row shown is the best-fit time lag (which is consistent with that in Table S3A). Despite the lower value for the adjusted-R^2 as compared to the fit to Eq. (1), the parameter estimates are significant, with p values of 1.55e-3, and 1.01e-4, for α and β respectively.

US coal	N	$logP_0$	std error	α	std error	β	std error	adj-R^2
t_R=0, t_C=0	33	-5.49	0.53	0.18	0.03	0.46	0.03	0.995
t_R= -1, t_C=-1	32	-4.67	0.42	0.11	0.02	0.45	0.02	0.996
**t_R=-1, t_C= 0**	**32**	**-4.77**	**0.34**	**0.18**	**0.02**	**0.43**	**0.02**	**0.997**
t_R= 0, t_C=-1	32	-4.99	0.49	0.13	0.04	0.46	0.03	0.995
t_R=-2, t_C= -2	31	-4.12	0.39	0.05	0.02	0.45	0.02	0.996

Table S4A: Results of applying Eq. (1) in the main text to coal technologies. Production in this case refers to energy generation (rather than capacity). Patents, production and R&D investments are for the US only. The highlighted row indicates the best-fit parameters used in Figure 4 of the main text. p-values for best fit parameters (highlighted) are 1.67e-14, 1.74e-12, < 2e-16.

US coal	N	α	std error	β	std error	adj-R^2
**t_R=-1, t_C=0**	**32**	**0.29**	**0.01**	**0.68**	**0.08**	**0.949**

Table S4B: Results of applying Eq. (2) in the main text to the first difference of the logs of cumulative US coal patents, R&D funding and energy generation. These time series pass the Dickey-Fuller test of stationarity. The row shown is the best-fit time lag (which is consistent with that in Table S4A). The parameter estimates are significant, with p values of 1.67e-23, and 1.74e-9, for α and β respectively.

Solar<br>RDD only	N	$logP_0$	std error	α	std error	adj-R^2
t_R= 0	34	-1.05	0.73	1.03	0.08	0.830
t_R= -1	33	-0.04	0.72	0.93	0.08	0.810

Table S5A: Best fit parameters for solar technologies applying Eq. (1) from the main paper but including R&D only (not production). Note that the fits are worse than those of Table S2A, suggesting that market growth of the technology is an essential ingredient stimulating patents and knowledge creation.

Solar<br>RDD only	N	α	std error	adj-R^2
t_R= 0	34	0.51	0.05	0.161
t_R= -1	33	0.45	0.05	-0.096

Table S5B: Best fit parameters for solar technologies applying Eq. (2) from the main paper but including R&D only (not production). Note that the fits are worse than those of Table S2B, indicating the importance of market growth.

Wind RDD only	N	$logP_0$	std error	α	std error	adj-R^2
t_R= 0	26	-4.31	0.80	1.53	0.10	0.902
t_R= -1	25	-3.51	0.87	1.45	0.11	0.879

Table S6A: Best fit parameters for wind energy applying Eq. (1) from the main paper, including R&D only. Note that the fits are worse than those of Table S3A.

Wind RDD only	N	α	std error	adj-R^2
t_R= 0	26	1.06	0.14	-1.550
t_R= -1	25	0.86	0.16	-4.70

Table S6B: Best fit parameters for wind energy technologies applying Eq. (2) from the main paper, including R&D only. The fits are worse than those of Table S3B.

US coal RDD only	N	$logP_0$	std error	α	std error	adj-R^2
t_R= 0	33	2.27	0.16	0.60	0.02	0.959
t_R= -1	32	2.93	0.16	0.52	0.02	0.952

Table S7A: Best fit parameters for coal energy technologies in the US applying Eq. (1) from the main paper and including R&D only (not production). Note that though the fits are worse than those of Table S4A, the difference is smaller than for other technologies.

US coal RDD only	N	α	std error	adj-R^2
t_R= 0	33	0.42	0.03	0.79
t_R= -1	32	0.37	0.02	0.82

Table S7B: Best fit parameters for US coal applying Eq. (2) from the main paper including R&D only (not production). Note that the fits are worse than those of Table S4B.

4. Supporting figures

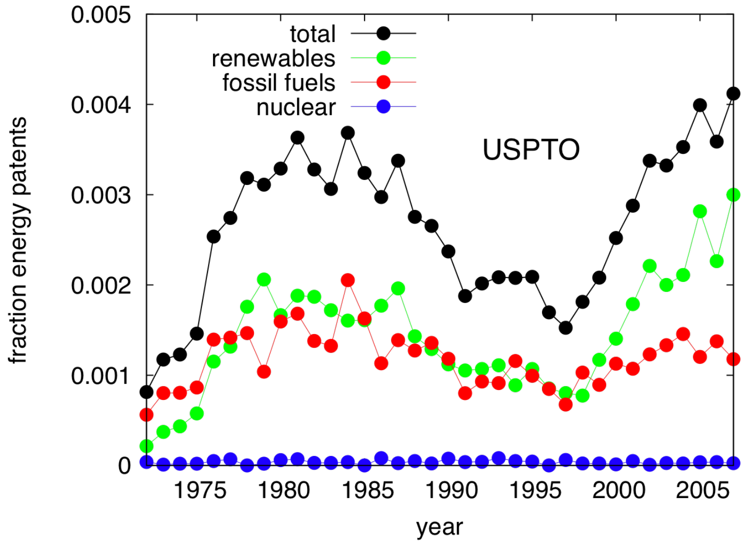

Figure S1: Temporal trends for the fraction of all patents accounted for by energy technologies filed in the USA (USPTO). This fraction was computed using US patents granted (as reported elsewhere in this study) over the total number of US applications for all sectors, with a time lag of two years (the average time between application and granting a patent, sometimes referred to as the average pendency time). These trends indicate that energy patents are growing faster than overall patenting rates, which themselves have been increasing over time due to high growth rates in highly innovative sectors such as the semiconductor, computer technology, biotechnology, and medical technology industries. The data shows that the fractional high rates of energy patenting are mostly due to activity in renewable energy technologies.

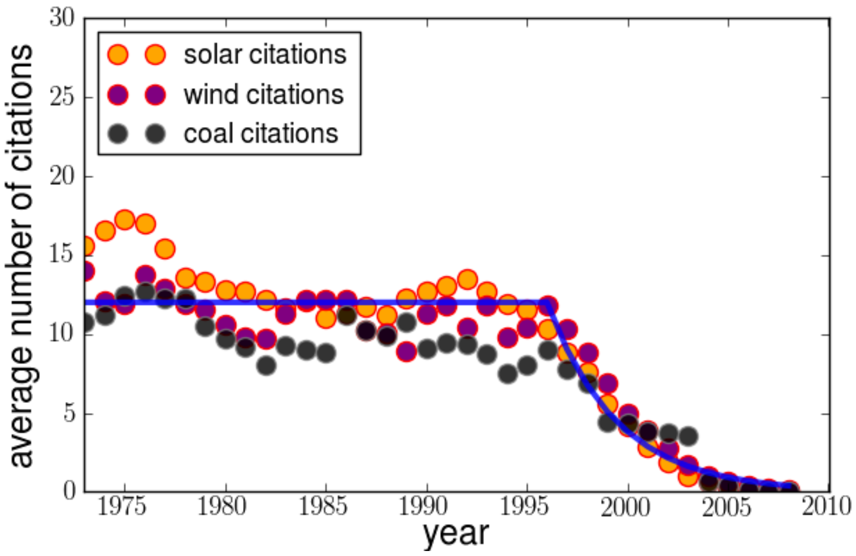

Figure S2: **Citation counts for patents submitted in solar (photovoltaic), coal and wind technologies as a function of submission year.** The decay in number of citations since 1996 is well modeled by an exponential with temporal constant τ=3.5 years (solid blue line) and is common to all technologies. This decay is due to the period necessary for any patent to accumulate citations.

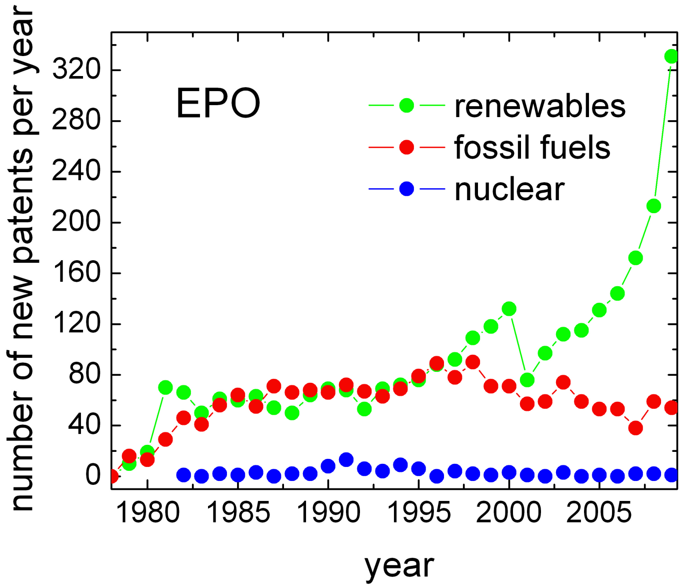

Figure S3a: Temporal trends for patents filed in the European Patent Office (EPO) for nuclear, fossil and renewable technologies (different colors). Note the earlier high relative proportion of innovation in renewables compared to other nations, and the recent drop in fossil fuel technologies.

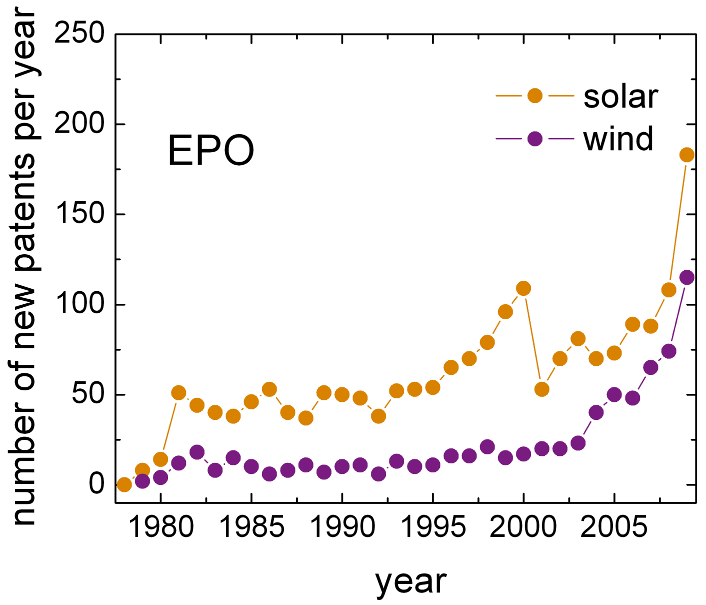

Figure S3b: Temporal trends for patents filed in the European Patent Office (EPO) for solar and wind energy technologies.

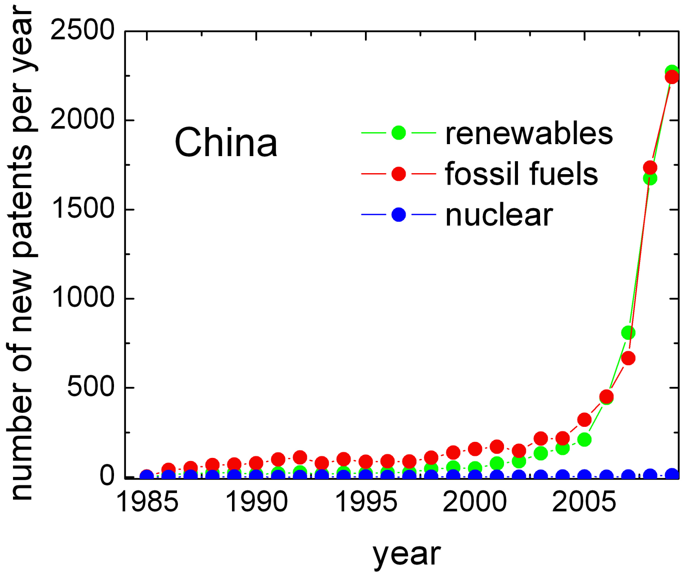

Figure S4a: Temporal trends for patents filed in China for nuclear, fossil and renewable technologies (different colors). Note the rise in fossil fuel technologies, but the even faster increase in renewables.

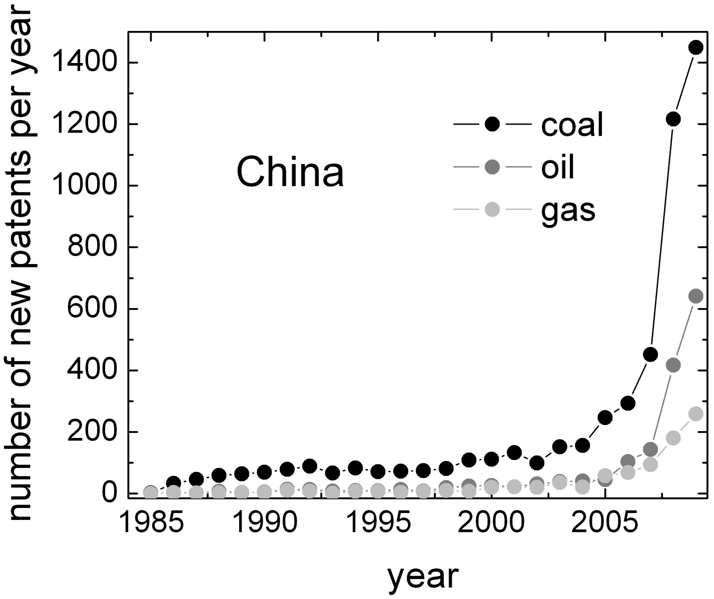

Figure S4b: Temporal trends for patents filed in China for coal, oil and gas energy technologies.

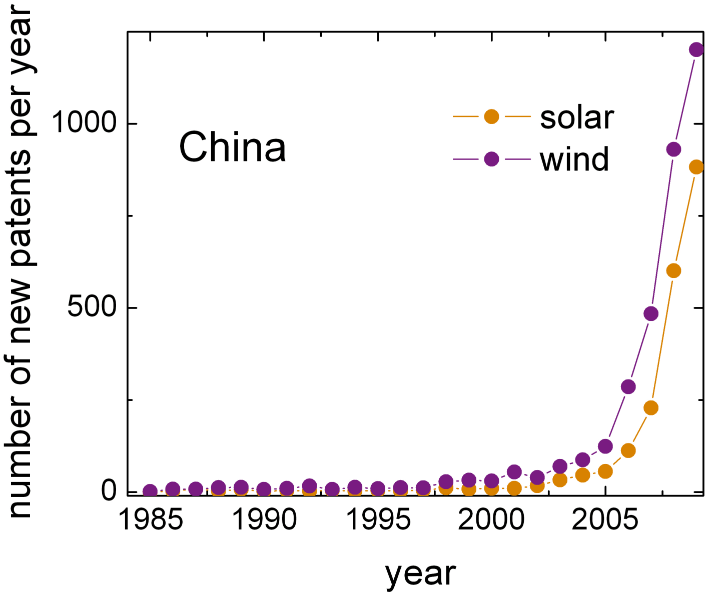

Figure S4c: Temporal trends for patents filed in China for solar and wind energy technologies.

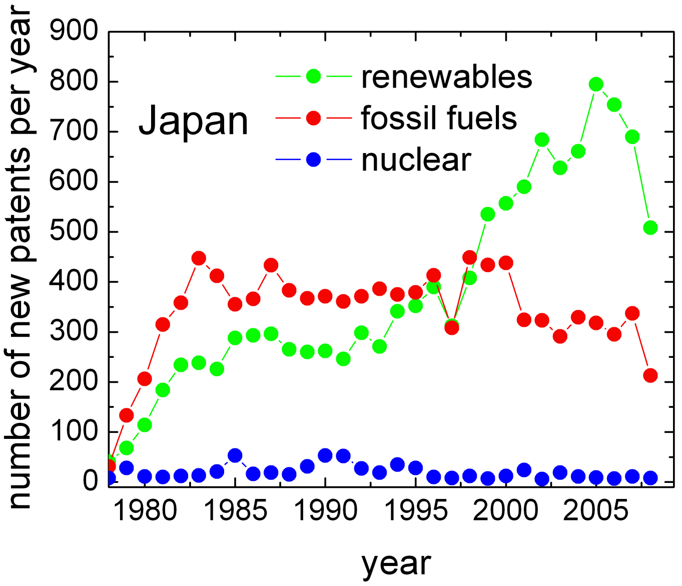

Figure S5a: Temporal trends for patents filed in Japan for nuclear, fossil and renewable technologies (different colors). Note the recent decline in annual patents, and the numbers in relation to Figure S4.

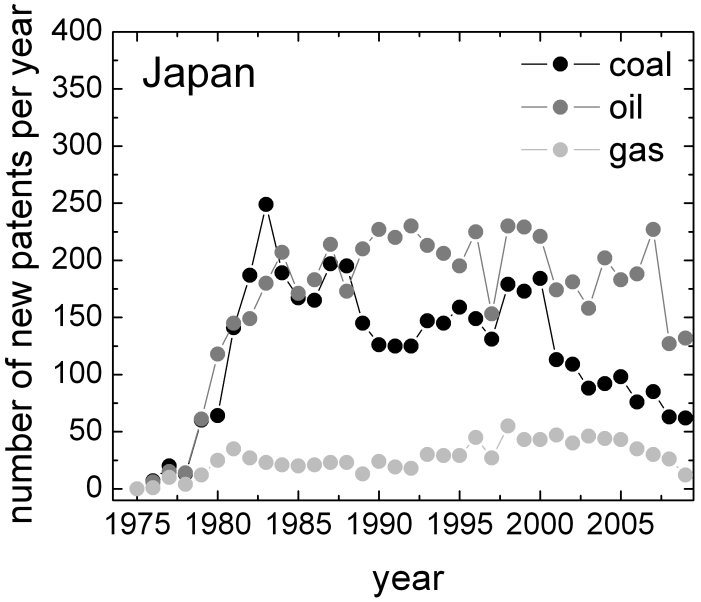

Figure S5b: Temporal trends for patents filed in Japan for coal, oil and gas energy technologies.

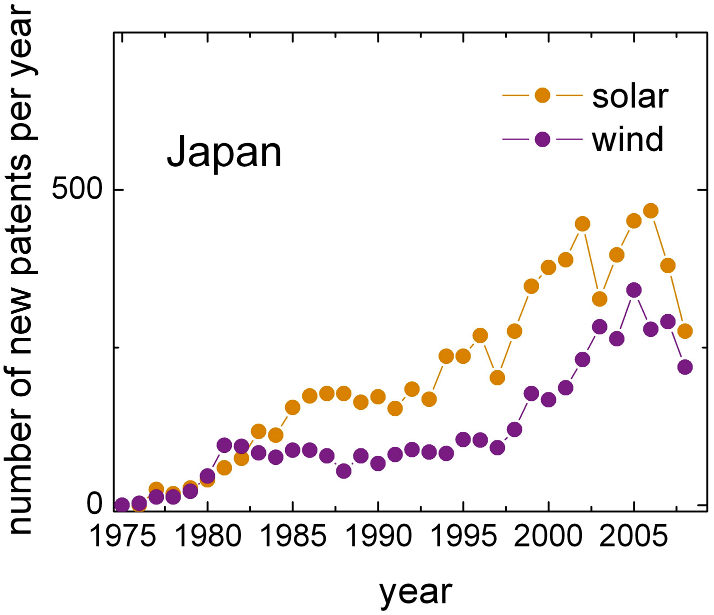

Figure S5c: Temporal trends for patents filed in Japan for solar and wind energy technologies.

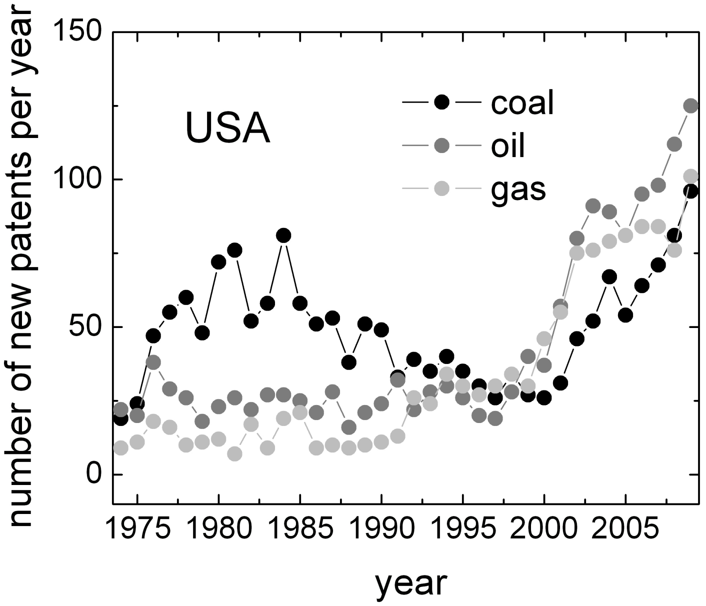

Figure S6a: Temporal trends for patents filed in the US for coal, oil and gas energy technologies.

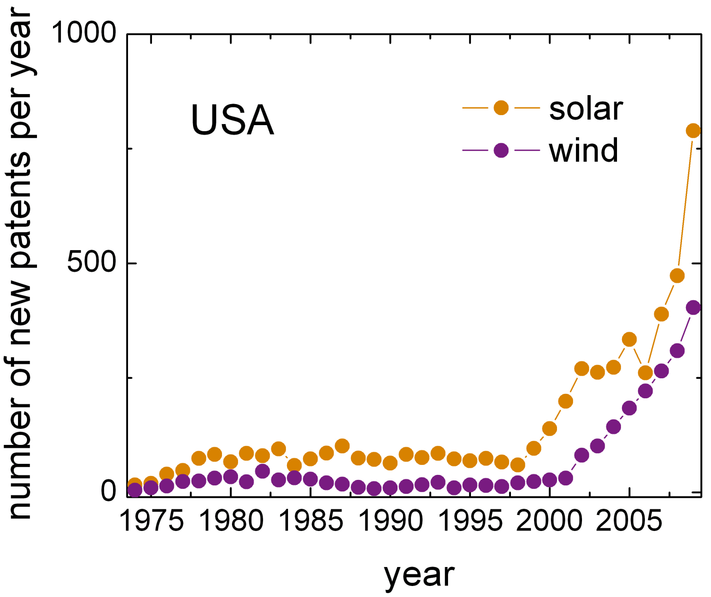

Figure S6b: Temporal trends for patents filed in the US for solar and wind energy technologies.

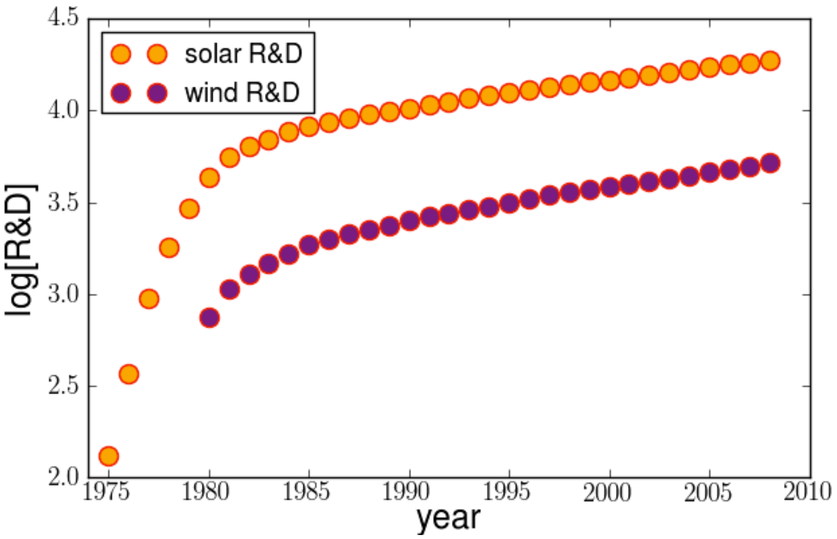

Figure S7: Solar (photovoltaics) and wind cumulative public R&D investment ($2009 million) for total of IEA nations.

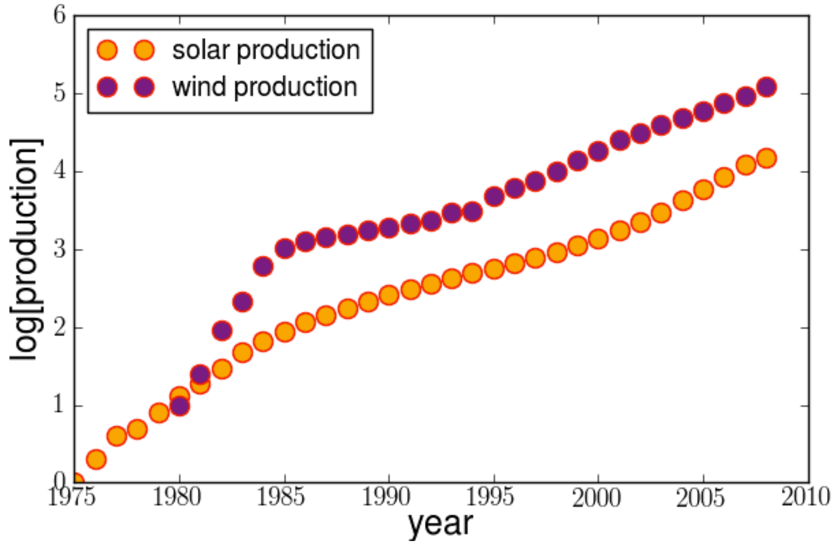

Figure S8: Photovoltaics and wind cumulative worldwide production (MW). Note the uptick in rates of production for wind in the mid 1990s and solar in the early 2000s.

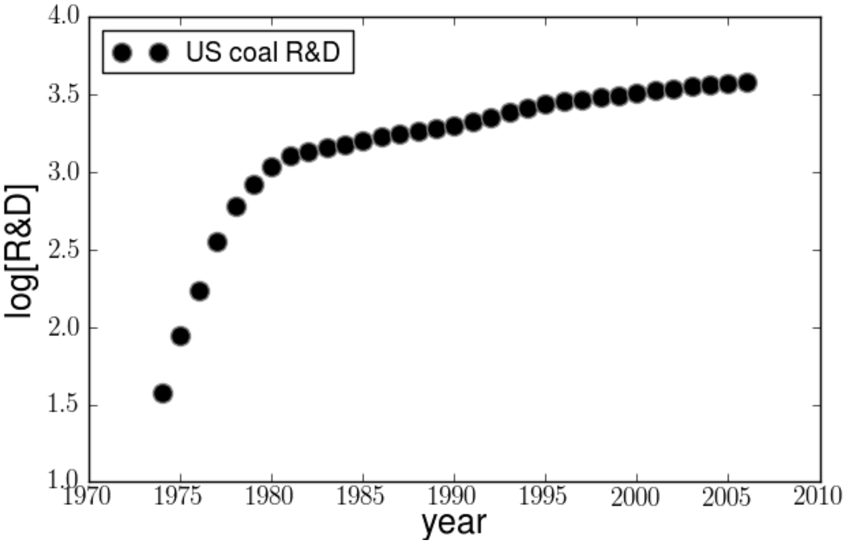

Figure S9: Cumulative public R&D investments in coal based technologies in the US ($2009 million). The focus on the US is motivated by more limited knowledge spillover in the case of coal than solar and wind (higher installation and operating costs).

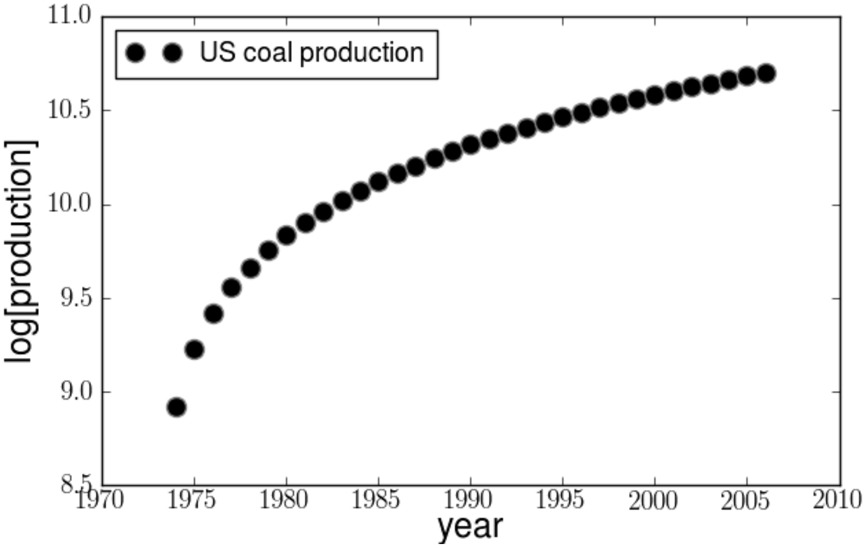

Figure S10: Cumulative energy production in the US from coal based technologies (MWh). The focus on electricity (units of energy produced) in this case is motivated by the significant costs incurred in the conversion of coal-stored energy to electricity, and the potential for innovation to reduce these costs (8).

References

1. Margolis RM & Kammen DM (1999) Underinvestment: The Energy Technology and R&D Policy Challenge. *Science* 285(5428):690-692.
2. Margolis RM & Kammen DM (1999) Evidence of under-investment in energy R&D in the United States and the impact of Federal policy - Basic Science and Technological Innovation. *Energy Policy* 27:575-584.
3. IEA (2009) IEA Energy Statistics, R&D Database. (International Energy Agency).
4. Nemet GF & Kammen DM (2007) U.S. energy research and development: Declining investment, increasing need, and the feasibility of expansion. *Energy Policy* 35(1):746-755.
5. EurObserv'ER & The European Commission (2011) Wind Power Barometer.
6. EurObserv'ER & The European Commission (2011) Solar Power Barometer.
7. World Intellectual Property Organization (2008) World Patent Report: A Statistical Review.
8. McNerney J, Farmer JD, & Trancik JE (2011) Historical costs of coal-fired electricity and implications for the future. *Energy Policy* 39:3042-3054.
9. Griliches Z (1990) Patent Statistics as Economic Indicators: A Survey. *Journal of Economic Literature* 28:1661-1707.
